# Frequent detection of CXCR4-using viruses among Brazilian blood donors with HIV-1 long-standing infection and unknown clinical stage: Analysis of massive parallel sequencing data

**DOI:** 10.1016/j.dib.2015.12.008

**Published:** 2015-12-17

**Authors:** Rodrigo Pessôa, Sabri S. Sanabani

**Affiliations:** Department of Pathology, Hospital das Clínicas, School of Medicine, University of São Paulo, São Paulo, Brazil

**Keywords:** Human immunodeficiency virus type 1, Viral tropism, Massively parallel sequencing

## Abstract

The determination of viral tropism is critically important and highly recommended to guide therapy with the CCR5 antagonist, which does not inhibit the effect of X4-tropic viruses. Here, we report the prevalence of HIV-1×4 HIV strains in 84 proviral DNA massively parallel sequencing “MPS” data from well-defined non-recently infected first-time Brazilian blood donors. The MPS data covering the entire V3 region of the *env* gene was extracted from our recently generated HIV-1 genomes sequenced by a paired-end protocol (Illumina). Of the 84 MPS data samples, 63 (75%) were derived from donors with long-standing infection and 21 (25%) were lacking stage information. HIV‐1 tropism was inferred using Geno2pheno (g2p) _[454]_ algorithm (FPR=1%, 2.5%, and 3.75%). Among the 84 data samples for which tropism was defined by g2p_2.5%_, 13 (15.5%) participants had detectable CXCR4-using viruses in their MPS reads. Mixed infections with R5 and X4 were observed in 11.9% of the study subjects and minority X4 viruses were detected in 7 (8.3%) of participants. Nine of the 63 (14.3%) subjects with LS infection were predicted by g2p _2.5%_ to harbor proviral CXCR4-using viruses. Our findings of a high proportion of blood donors (15.5%) harboring CXCR4-using viruses in PBMCs may indicate that this phenomenon is common. These findings may have implications for clinical and therapeutic aspects and may benefit individuals who plan to receive CCR5 antagonists.

## 1 Introduction

Human immunodeficiency virus type 1 (HIV-1) infects cells through interaction with the CD4 receptor and one of two chemokine coreceptors, either CCR5 or CXCR4 [1]. Approximately 80–90% of recently infected and treatment-naïve HIV-1 patients have a virus that uses the CCR5 coreceptor (R5 virus) [2], while the CXCR4 coreceptor-using virus (X4 virus) and dual-tropic viruses that use both CCR5 and CXCR4 (R5×4) emerge and coexist in nearly half of non-treated subtype B and D infected patients in advanced disease stages but are found less often in subtype A and C infected individuals [3]. The development of the CCR5 coreceptor blocker maraviroc, which has exclusive activity against R5 viruses, has attracted considerable attention in co-receptor affinity or tropism [4]. It is strongly recommended that we determine co-receptor tropism before initiating treatment with entry inhibitors because these drugs have no effect on X4 populations [5].

One of the principal approaches to assess HIV-1 coreceptor use is the genotypic assay, which infers viral tropism from sequence information of the third hypervariable (V3) loop of gp120 in the envelope protein. This approach comprises two steps: the sequencing assay, and the prediction method interpreting the sequence data. Sequencing is usually based on the conventional “bulk” Sanger sequencing method. This approach is neither ideal for sequencing DNA that contains nucleotide mixtures (*quasispecies*) nor DNA with mutations being present in at least 15–20% of the viral population. Most of these limitations have now been overcome by the introduction of next generation sequencing. This technology allows, for instance, sequencing not only single clones but also viruses prevalent in minor populations [6], [7], [8]. Despite the larger number of methods developed for predicting HIV-1 coreceptor use, only a small number of these algorithms are implemented as web-service. Among the available web-tools, geno2-pheno co-receptor (g2p) is the most used algorithm applied to Support Vector Machines (SVMs) to infer coreceptor use. Unlike other methods, gen2pheno, for example, can handle massively parallel sequencing [MPS] data and allows for changing the specificity-level of the prediction method.

Our group has recently reported the prevalence of co-receptor tropism of the archived viral strains at the time of primary infection using MPS data from 45 recently infected Brazilian first-time blood donors [9]. In continuation with our previous study, here, we assessed the prevalence of virus co-receptor use in MPS data from 84 Brazilian first-time blood donors with long-standing infection (LS, *n*=63) and others lacking the stage information (*n*=21). Although plasma HIV-1 RNA has been widely used to determine the viral tropism the proviral PBMC DNA sequence can contain a variety of multiple archived genomes that are not present in plasma. This, combined with the stability of DNA compared with RNA, and the fact that HIV DNA recovered from the proviral compartment can reliably be used as an alternative to RNA tropism testing [9], [10], [11], [12], [13] influenced our decision to use proviral DNA in this study. No data, to the best of our knowledge, have been published so far for genotypic tropism analysis in such a specific patient group.

## 2 Methods

The MPS data used in the present study were derived from 84 HIV-1 proviral near full-length genomes (NFLG) and larger partial fragment sequences with predetermined subtypes. Of these, 63 (75%) were derived from donors with LS infection and 21 (25%) were lacking stage information. The genetic subtype was determined by Illumina ultra-deep sequencing technology of the proviral near-full length genomes (submitted for publication). Samples with long-standing infection were previously classified by less-sensitive or “detuned” enzyme immunoassay (Vironostika HIV-1 MicroElisa; bioMérieux, Durham, NC) or an LS chemiluminescent immunoassay (Vitros HIV-1/2 Assay; Ortho Diagnostics, Rochester, NY) [14]. None of the participants received antiviral treatment previously. All study subjects provided written informed consent. The study was approved by the local ethical review committee of participating institutions as well as the REDS-II collaborating centers (Blood Systems Research Institute/University of California at San Francisco, San Francisco, CA) and data coordinating center (Westat, Inc.) in the United States.

The genomic DNA used for the PCR analyses was extracted using the QIAamp blood kit (Qiagen GmbH, Hilden Germany) according to the manufacturer׳s instructions. The NFLGs from five overlapping fragments were obtained by PCR using the Platinum Taq DNA Polymerase High Fidelity (5 U/µl) (Invitrogen, Life Technologies, Carlsbad, CA) and determined by a previously reported method [15], [16]. The amplified DNA fragments from the nested PCR products were separated by gel electrophoresis and purified using Freeze ‘N Squeeze DNA Gel Extraction Spin Columns (Bio-Rad, Hercules, CA, USA). Each purified amplicon was quantified using Quant-IT HS reagents (Invitrogen, Life Technologies, Carlsbad, CA), and all five amplicons from a single viral genome were pooled together at equimolar ratios.

Sequencing libraries were prepared as described previously [17], [18], [19]. Briefly, 1  ng of each sample amplicon pool was used in a fragmentation and tagmentation reaction mix using the DNA sample prep kit according to the manufacturer׳s protocol (Nextera XT, Illumina, San Diego, CA). After neutralization of the fragmented DNA, a light 12-cycle PCR was performed with Illumina Ready Mix to add Illumina flowcell adaptors, indexes, and common adapters for subsequent cluster generation and sequencing. Amplified DNA libraries were then purified using Agencourt AMPure XP beads (Beckman Coulter, Brea, CA), which excluded very short library fragments. Finally, all libraries were pooled and loaded on an Illumina MiSeq for paired-end 250 sequencing.

Validated fastq files from each viral genome were de novo assembled into contiguous sequences and annotated with CLC Genomics Workbench Version 7.0.4 (CLC Bio, Aarhus, Denmark) with default settings and were additionally assembled using Velvet implemented in the Sequencher program 5.2 (Gene Code Corp., Ann Arbor, MI). The contiguous genomic sequence from each NFLGs and larger fragments of virus strain was extracted from the assembly and used for further analysis.

In this study, a sub-library of the *env* V3 population sequence derived from each sample was created by mapping the raw MPS short reads to their corresponding V3 consensus sequence (Sequences positions: 210–315 [GenBank accession no. K03455] in standard reference HXB2) using the CLC Genomics Workbench version 7.0.4 (CLC Bio, Aarhus, Denmark). To avoid artificial generation of *in silico* chimeras through assembly and to evade inflating the diversity estimates of the V3 region, the analysis was restricted to individual paired-end reads that encompass the complete V3 region from each dataset. Only samples with a depth of average coverage of ≥500x were considered for the analysis. Prior to the determination of viral tropism, the MPS data were filtered out by the presence of frame shifts, stop codons, and base-call ambiguity.

HIV-1 co-receptor tropism was assessed from the filtered V3 MPS data using the g2p _[454]_ tool and classified as X4 when there were more than 2% of the sequences with g2p false positivity rate (FPR) cutoffs of 3.75%, 2.5% and 1%. More detailed analysis was performed at FPR set at 2.5% to increase the X4 detection sensitivity without affecting the specificity. The values of the FPR used here are based on several studies that indicate the capacity of g2p algorithms to provide reliable discrimination between R5 and X4 sequences when FPR is set at lower values [20]. In this study, a minority variant was defined as a variation detected at ≥2% and <20% of the virus MPS reads, corresponding to those mutations that cannot be established using the conventional sequencing technology. The 2% cutoff was established because it was found to be optimal to predict the clinical response [21].

### 2.1 Nucleotide sequence accession numbers

The sequencing data have been uploaded to Zenodo. 10.5281/zenodo.32950

## 3 Results

Phylogenetic analysis of the NFLGs and larger partial fragment that cover the *env gp 120* region were performed in HIV-1 infected donors with LS infection (*n*=193) and an unknown clinical stage (n=64) (submitted manuscript). After removal of scaffolding reads not covering the complete V3 region from partial fragments and exclusion of MPS data with low coverage reads (<500x) and poor quality reads, the number of samples was dropped to 84 samples and these were considered for analysis. The coverage after mapping of the sample to its corresponding consensus sequence varied among the patients showing an overall median sequence depth of 1030 (range: 509-6883). Of the 84 investigated subjects, 69 (82.1%) belonged to individuals carrying HIV-1 subtype B, 11 (13.4%) subclade F1, and 4 (4.8%) subtype C. The g2p algorithm_1%_ predicted the occurrence of X4 strains in >94% of generated MPS reads of three (3.6%) participants (coverage depth range: 636–2300) (Table 1). No minority variants (X4 viruses at a frequency below 20%) were observed under this algorithm. At a g2p algorithm _2.5%_ and _3.75%_, the CXCR4-using viruses were predicted in 13 (15.5%) and 20 (23.8%) subjects (coverage depth range: 564–2937), respectively. At the setting of g2p algorithm _2.5%_, three participants (3.7%) had detectable X4 viruses in >99% of their MPS reads (coverage depth range: 636–2300). Furthermore, seven (8.3%) subjects were found to harbor minority X4 viruses at a frequency rate below 20% within their viral population (coverage depth range: 564–1868). Besides the seven participants with X4 minority variants, our analysis revealed other three other participants namely: 10BR_PE044, 10BR_PE106, and 10BR_PE024 to harbor CXCR4-using viruses at a frequency of 35.5%, 57.9%, and 65.1%, respectively. Taken together, these results indicate a high rate (11.9%) of R5×4 mixed infection in the studied population. Of note, all the 13 MPS data found to contain CXCR4-using viruses were characterized by phylogenetic analysis of the V3 region to belong to subtype B viruses. Analysis of the MPS data from the 63 subjects with LS infection revealed that 9 (14.3%) participants had CXCR4-using proviruses (coverage depth range: 564–2937). Among the 63 subjects, the minority variants and R5×4 viruses were detected in 4 (6.3%) and 6 (9.5%) subjects, respectively.

## 4 Discussion

Recently we reported our initial findings of the prevalence of coreceptor tropism of the archived strains at the time of primary infection using a total of 45 MPS data from HIV-1 recently infected Brazilian first-time blood donors [9]. In the present study, we expanded our previous work by determining the prevalence of CXCR4-using viruses in MPS data from a total of 84 Brazilian first-time blood donors consisting of 63 subjects with LS infection and 21 with unknown stage information. Some studies have reported the presence of X4 strains in recent HIV-1 seroconverter Spanish, Brazilian and French subjects [16], [22] and in drug-naive chronically HIV-infected individuals [23], in immunosuppressed patients with a shorter history of viremia suppression [24], in patients failing antiretroviral therapy [25], and in patients with detectable HIV-1 subtype B RNA receiving highly active antiretroviral therapy [26]. However, to our knowledge, only one relevant study to date has explored the coreceptor use in MPS data but from recently infected and therapy naive first-time Brazilian blood donors [9].

Among the 84 MPS data analyzed by g2p algorithm_2.5%_, the CXCR4-using viruses were predicted in 13 (15.5%). Also, a higher rate (14.3%) of CXCR4-using viruses was observed among the LS group in this study. These results were comparable to our previous study [9], which reported a relatively high frequency (13.3%) of CXCR4-using viruses in 45 HIV-1 recently infected donors, despite the fact that they were based on different FPR algorithms (>3.5% and >2.5%). Our results can also be compared with those reported in drug-naive chronically HIV-infected individuals [27] and in suppressed patients with a shorter history of viremia suppression [24]. In contrast, our prevalence estimates of CXCR4-using viruses are nearly five times higher than those found among recently infected men having sex with men in the USA [28] and almost half the prevalence rate reported in recently infected Brazilian subjects [16]. Factors like size and type of samples, the sequencing method, the FPR cutoff, stage of HIV infection (primary vs chronic infection), and prediction algorithms used may have contributed to these differences. Studies like ours that based on MPS data generally detect more CXCR4 variants compared to the conventional genotypic sequencing technologies. For instance, of the 84 studied subjects, our study was able to detect seven subjects (8.3%) with X4 minority variants (fewer than 20% abundance) that would likely have been missed if standard population-based sequencing data had been used alone. Thus, the application of deep sequencing technology has significantly improved the prediction of HIV tropism as has been reported in previous studies [16], [21].

Detection of R5×4 mixed infection in this study could be either the result of direct transmission of both variants, successive infections within a short timeframe, or a rapid switch from CCR5-using to CXCR4-using virus shortly after transmission. The hypothesis of direct transmission of both variants could result from a stochastic process as has been suggested previously [29].

Because CXCR4-using viruses are more pathogenic than R5 viruses [30], larger studies are needed to confirm the negative impact of these variants on the subsequent evolution of HIV-1 disease and to investigate the efficiency of these variants to influence the patient׳s response to CCR5 antagonists.

We acknowledge that our study had a number of limitations that should be highlighted. The most important limitation is that the assessment of HIV tropism was limited to sequence-based algorithms rather than phenotypic methods. Although phenotypic assays still have an edge over genotypic methods, Ultra-deep sequencing data prove to be highly concordant with phenotype data for determining HIV-1 co-receptor use during primary HIV infection [31] and can reliably be used to determine viral tropism with better results in PBMC than in plasma samples [32]. Other limitations include small sample size and relatively low sequencing coverage (500X). Also, our V3 MPS data were derived from specific groups of HIV-1 infected first-time blood donors and the results may not reflect the prevalence in the general populations.

Despite these limitations, our findings show a relatively high frequency of CXCR4-using variants in long standing HIV-1 infected blood donors. An independent study will be needed to explore the clinical relevance of these variants in light of the clinical progression, pathogenesis, and therapeutic approach.

## 5 Conclusions

Our data on the prevalence HIV-1×4 strains are important for therapy planning and draw attention to the need to adequately monitor the prevalence of these variants in other clinical settings.

## Competing interests

The authors declare that they have no competing interests.

## Authors׳ contributions

Conceived and designed the experiments: SSS. Analyzed the data: RP and SSS. Wrote the manuscript: SSS.

## Figures and Tables

**Table 1:** Tropism predictions of CXCR4-using viruses at FPR cut off of 1%, 2.5%, and 3.75% obtained from massively parallel sequencing data.

Sample number	Quality reads	Predicted X4 using at FPR%^1^ cut-off			V3 subtype	NFLG^2^ subtype	Clinical stage
1	2.5	3.75
10BR_PE004	750	0%	0.4%	2.2%	B	CRF70_BF1	Lack information
10BR_PE003	2892	0%	0.6%	2.6%	B	B	Lack information
10BR_PE071	1117	0%	0.8%	2.7%	B	CRF71_BF1	Long-standing infection
10BR_RJ042	613	0.1%	0.8%	5%	B	B	Long-standing infection
10BR_RJ111	906	0.1%	0.8%	2.7%	B	B	Lack information
10BR_RJ085	864	0%	1.1%	2.5%	B	B	Lack information
10BR_PE030	669	0.1%	1.3%	2%	B	B	Long-standing infection
10BR_PE019	564	0.1%	2.1%	2.6%	B	B	Long-standing infection
10BR_PE045	1824	0.4%	2.1%	5%	B	B	Long-standing infection
10BR_RJ101	1868	0.1%	2.1%	2.4%	B	B	Lack information
10BR_RJ110	949	0.1%	2.1%	2.1%	B	B	Lack information
10BR_RJ094	1104	0.1%	2.2%	3.7%	B	BF1	Long-standing infection
10BR_RJ079	1332	0.1%	3.8%	8.8%	B	B	Lack information
10BR_PE044	753	1.1%	35.5%	90.3%	B	B	Lack information
10BR_PE052	948	0.1%	5.5%	66.6%	B	B	Long-standing infection
10BR_PE106	1358	0.1%	57.9%	98.9%	B	B	Long-standing infection
10BR_PE024	2937	1.4%	65.1%	65.1%	B	B	Long-standing infection
10BR_SP013	636	94.9%	99.6%	99.6%	B	BF1	Long-standing infection
10BR_PE096	2300	98.2%	99.7%	99.7%	B	B	Long-standing infection
10BR_PE059	1224	96.7%	99.7%	99.9%	B	BF1	Long-standing infection

1 False positivity rate.

2 Near full-length genomes.
